# Supplementary material for: Age-related decline in blood-brain barrier function is more pronounced in males than females in parietal and temporal regions
Source: eLife. 2024 Nov 4;13:RP96155. doi: 10.7554/eLife.96155 (PMC11534331; doi:10.7554/eLife.96155)
Supplement: Supplementary file 1. — (a) Analysis of age and sex-dependent kw variations in 14 brain regions using MARS. Threshold ages when kw starts declining have been identified. The age ×sex interaction terms being negative suggest a more pronounced decline in kw in males compared to females. For females, the post-threshold age kw decline slope is presented with 95% confidence intervals (CI) and P values. The male kw decline slopes were estimated from the female slope and the age ×sex interaction. Entries labeled 'N.A. (N.S.)' indicate non-significant changes. (b) Analysis of age and sex dependent CBF variations in 14 brain regions using MARS. Threshold ages where CBF slope changes occur were identified. A negative age ×sex interaction term was detected only in the hippocampus, suggesting a more pronounced CBF decline in males compared to females. In other brain regions, the rate of CBF decline with age is largely similar between males and females, although males consistently exhibit lower CBF. For both males and females, the CBF decline slopes before and after the threshold age are presented with 95% confidence intervals (CIs) and P values. (c) Analysis of age and sex dependent ATT variations in 14 brain regions using MARS. Threshold ages where ATT slope changes occur were identified. The rate of ATT increase with age is largely similar between males and females, although males consistently exhibit longer ATT. For both males and females, the ATT increase slopes before and after the threshold age are presented with 95% confidence intervals (CIs) and P values. (d) Voxel-wise analysis of age trend in kw. Brain regions with significant negative correlations between kw and age with corresponding location (AAL template), cluster size, T values, and P values. (e) Voxel-wise analysis of age and sex effect in kw, CBF, and ATT. Brain regions with significant age ×sex effects in kw, CBF, and ATT with corresponding locations (AAL template), cluster sizes, T values, and P values. (f) Association between kw an [file elife-96155-supp1.docx]

**Supplementary file 1a**

| **Brain regions** | **Threshold age (years)** | **Age×Sex (F: 0, M: 1)**  (**Estimate** [95% CI]; **P value**) | **Slope for female**  (**Estimate** [95% CI]; **P value**) | **Slope for Male**  (**Estimate**) |
| --- | --- | --- | --- | --- |
| **Gray matter** | 62 | N.A. (N.S.) | **-0.82** [-1.35, -0.28]; **0.003** | **-0.82** |
| **White matter** | 62 | N.A. (N.S.) | **-0.87** [-1.42, -0.33]; **0.002** | **-0.87** |
| **Frontal lobe** | N.A. | N.A. (N.S.) | N.A. (N.S.) | N.A. |
| **Temporal lobe** | N.A. | **-0.22** [-0.34, -0.1]; **0.001** | N.A. (N.S.) | **-0.22** |
| **Parietal lobe** | 19 | **-0.2** [-0.35, -0.04]; **0.01** | **-0.33** [-0.76, -0.3]; **0.01** | **-0.53** |
| **ACC** | 36 | N.A. (N.S.) | **-0.35** [-0.59, -0.11]; **0.005** | **-0.35** |
| **PCC** | 19 | N.A. (N.S.) | **-0.41** [-0.63, -0.18]; **0.001** | **-0.41** |
| **Precuneus** | 19 | N.A. (N.S.) | **-0.36** [-0.57, -0.16]; **0.001** | **-0.36** |
| **Caudate** | 62 | N.A. (N.S.) | **-0.83** [-1.41, -0.24]; **0.006** | **-0.83** |
| **Putamen** | N.A. | N.A. (N.S.) | N.A. (N.S.) | N.A. |
| **Amygdala** | N.A. | N.A. (N.S.) | N.A. (N.S.) | N.A. |
| **Hippocampus** | 19 | **-0.14** [-0.26, -0.02]; **0.02** | **-0.12** [-0.43, -0.09]; **0.003** | **-0.26** |
| **PHG** | N.A. | **-0.15** [-0.26, -0.04]; **0.006** | N.A. (N.S.) | **-0.15** |
| **MTL** | N.A. | **-0.15** [-0.26, -0.04]; **0.008** | N.A. (N.S.) | **-0.15** |

**Supplementary file 1a: Analysis of age and sex dependent kw variations in 14 brain regions using MARS.** Threshold ages when kw starts declining have been identified. The age×sex interaction terms being negative suggest a more pronounced decline in kw in males compared to females. For females, the post-threshold age kw decline slope is presented with 95% confidence intervals (CI) and P values. The male kw decline slopes were estimated from the female slope and the age×sex interaction. Entries labeled 'N.A. (N.S.)' indicate non-significant changes.

**Supplementary file 1b**

| **Brain regions** | **Threshold age (years)** | **Sex effect (F: 0, M: 1)**  (Estimate (95% CI); P value) | **Slope for female and male**  (**Estimate** [95% CI]; **P value**) | |
| --- | --- | --- | --- | --- |
|  |  |  | Before threshold age | After threshold age |
| **Gray matter** | 22 | **-8.91** [-11.94, -5.88]; **<0.001** | **-1.39** [-2.01, -0.77]; **<0.001** | **-0.39** [-0.47, -0.31]; **<0.001** |
| **White matter** | 22 | **-7.81** [-10.57, -5.06]; **<0.001** | **-1.35** [-1.92, -0.79]; **<0.001** | **-0.27** [-0.34, -0.2]; **<0.001** |
| **Frontal lobe** | 22 | **-8.43** [-11.60, -5.25]; **<0.001** | **-1.52** [-2.17, -0.87]; **<0.001** | **-0.41** [-0.5, -0.33]; **<0.001** |
| **Temporal lobe** | 22 | **-9.36** [-12.56, -6.16]; **<0.001** | **-1.4** [-2.06, -0.75]; **<0.001** | **-0.41** [-0.49, -0.32]; **<0.001** |
| **Parietal lobe** | 22 | **-10.77** [-14.11, -7.43]; **<0.001** | **-1.56** [-2.25, -0.88]; **<0.001** | **-0.46** [-0.55, -0.37]; **<0.001** |
| **ACC** | 22 | **-8.01** [-11.44, -4.59]; **<0.001** | **-1.19** [-1.89, -0.48]; **0.001** | **-0.46** [-0.55, -0.36]; **<0.001** |
| **PCC** | 22 | **-10.97** [-15.16, -6.78]; **<0.001** | **-1.83** [-2.69, -0.97]; **<0.001** | **-0.46** [-0.58, -0.35]; **<0.001** |
| **Precuneus** | 22 | **-10.65** [-14.14, -7.16]; **<0.001** | **-1.77** [-2.49, -1.05]; **<0.001** | **-0.39** [-0.49, -0.3]; **<0.001** |
| **Caudate** | 29 | **-5.28** [-7.52, -3.04]; **<0.001** | **-0.82** [-1.09, -0.54]; **<0.001** | **-0.23** [-0.3, -0.16]; **<0.001** |
| **Putamen** | 22 | **-4.75** [-7.32, -2.17]; **<0.001** | **-1.01** [-1.54, -0.48]; **<0.001** | **-0.11** [-0.18, -0.04]; **0.001** |
| **Amygdala** | 52 | **-5.35** [-8.16, -2.53]; **<0.001** | **-0.15** [-0.29, -0.02]; **0.03** | **-0.33** [-0.51, -0.15]; **<0.001** |
| **PHG** | 52 | **-5.75** [-8.45, -3.04]; **<0.001** | **-0.13** [-0.26, 0]; **0.06** | **-0.43** [-0.6, -0.26]; **<0.001** |
| **MTL** | 52 | **-5.59** [-8.17, -3.02]; **<0.001** | **-0.17** [-0.29, -0.04]; **0.008** | **-0.37** [-0.53, -0.2]; **<0.001** |
|  |  |  |  |  |
| **Brain regions** | **Threshold age (years)** | **Age×Sex (F: 0, M: 1)**  (Estimate (95% CI); P value) | **Slope for female and male**  (**Estimate** [95% CI]; **P value**) | |
|  |  |  | Before threshold age | After threshold age |
| **Hippocampus** | 22 | **-0.1** [-0.14, -0.05]; **<0.001** | **F**: **-0.74** [-1.35, -0.32]; **0.002**  **M**: **-0.84** | **F**: **-0.17** [-0.34, -0.19]; **<0.001**  **M**: **-0.27** |

**Supplementary file 1b: Analysis of age and sex dependent CBF variations in 14 brain regions using MARS.** Threshold ages where CBF slope changes occur were identified. A negative age×sex interaction term was detected only in the hippocampus, suggesting a more pronounced CBF decline in males compared to females. In other brain regions, the rate of CBF decline with age is largely similar between males and females, although males consistently exhibit lower CBF. For both males and females, the CBF decline slopes before and after the threshold age are presented with 95% confidence intervals (CIs) and P values.

**Supplementary file 1c**

| **Brain regions** | **Threshold age (years)** | **Sex effect (F: 0, M: 1)**  (Estimate (95% CI); P value) | **Slope for female and male**  (**Estimate** [95% CI]; **P value**) | |
| --- | --- | --- | --- | --- |
|  |  |  | Before threshold age | After threshold age |
| **Gray matter** | 36 | **83.15** [37.19, 129.12]; **<0.001** | **N.A. (N.S.)** | **5.55** [3.83, 7.27]; **<0.001** |
| **White matter** | 36 | **73.93** [28.18, 119.68]; **0.002** | **N.A. (N.S.)** | **5.1** [3.39, 6.8]; **<0.001** |
| **Frontal lobe** | 36 | **84.99** [38.66, 131.32]; **<0.001** | **N.A. (N.S.)** | **5.67** [3.94, 7.4]; **<0.001** |
| **Temporal lobe** | 69 | **91.12** [42.21, 140.03]; **<0.001** | **5.49** [4.26, 6.72]; **<0.001** | **N.A. (N.S.)** |
| **Parietal lobe** | 43 | **N.A. (N.S.)** | **N.A. (N.S.)** | **6.9** [5.06, 8.74]; **<0.001** |
| **ACC** | 36 | **95.01** [43.55, 146.46]; **<0.001** | **N.A. (N.S.)** | **6.43** [4.89, 7.97]; **<0.001** |
| **PCC** | 36 | **N.A. (N.S.)** | **N.A. (N.S.)** | **5.55** [3.84, 7.25]; **<0.001** |
| **Precuneus** | 36 | **N.A. (N.S.)** | **N.A. (N.S.)** | **6.08** [4.53, 7.63]; **<0.001** |
| **Caudate** | 36 | **N.A. (N.S.)** | **N.A. (N.S.)** | **4.88** [3.48, 6.27]; **<0.001** |
| **Putamen** | 36 | **80.03** [31.87, 128.18]; **0.001** | **N.A. (N.S.)** | **5.27** [3.83, 6.71]; **<0.001** |
| **Amygdala** | 36 | **61.81** [16.47, 107.16]; **0.008** | **0.52** [-3.31, 4.34]; **0.79** | **5.0** [3.3, 6.69]; **<0.001** |
| **Hippocampus** | 52 | **N.A. (N.S.)** | **N.A. (N.S.)** | **6.5** [4.29, 8.71]; **<0.001** |
| **PHG** | 36 | **N.A. (N.S.)** | **N.A. (N.S.)** | **4.04** [2.78, 5.3]; **<0.001** |
| **MTL** | 52 | **N.A. (N.S.)** | **N.A. (N.S.)** | **6.45** [4.33, 8.57]; **<0.001** |

**Supplementary file 1c: Analysis of age and sex dependent ATT variations in 14 brain regions using MARS.** Threshold ages where ATT slope changes occur were identified. The rate of ATT increase with age is largely similar between males and females, although males consistently exhibit longer ATT. For both males and females, the ATT increase slopes before and after the threshold age are presented with 95% confidence intervals (CIs) and P values.

**Supplementary file 1d**

| **Brain regions** | **Location**  **(mm)** | **Cluster size (voxels)** | **T value** | **P value** |
| --- | --- | --- | --- | --- |
| **Prefrontal cortex** | (120, 122, 124) | 9026 | -2.55 | 0.022 |
| **Cingulate cortex** | (90, 160, 66) | 2543 | -2.47 | 0.024 |
| **Precuneus** | (92, 64, 118) | 2938 | -2.42 | 0.024 |
| **Lateral temporal lobe** | (108, 48, 82) | 8598 | -2.60 | 0.020 |
| **Occipital lobe** | (110, 48, 86) | 3967 | -2.60 | 0.020 |
| **Insula** | (48, 128. 74) | 1878 | -2.75 | 0.017 |

**Supplementary file 1d: Voxel-wise analysis of age trend in kw.** Brain regions with significant negative correlations between kw and age with corresponding location (AAL template), cluster size, T values and P values.

**Supplementary file 1e**

| **Measurement** | **Brain regions** | **Location**  **(mm)** | **Cluster size (voxels)** | **T value** | **P value** |
| --- | --- | --- | --- | --- | --- |
| **kw** | **Lateral prefrontal cortex** | (34, 118, 124) | 1055 | -2.22 | 0.031 |
|  | **Parietal lobe** | (128, 82, 122) | 1375 | -2.23 | 0.030 |
|  | **Lateral and medial temporal lobe** | (144, 66, 80) | 1517 | -2.24 | 0.030 |
| **CBF** | **Supra-marginal gyrus** | (144, 86, 100) | 584 | -2.27 | 0.029 |
|  | **Hippocampus** | (126, 110, 56) | 560 | -2.35 | 0.026 |
|  | **Frontal lobe** | (142, 142, 84) | 588 | -2.37 | 0.025 |
| **ATT** | **Supra marginal gyrus** | (122, 74, 122) | 1967 | 2.30 | 0.027 |
|  | **Posterior temporal lobe** | (144, 70, 82) | 4016 | 2.54 | 0.019 |
|  | **Calcarine sulcus** | (110, 56, 82) | 1531 | 2.33 | 0.026 |

**Supplementary file 1e: Voxel-wise analysis of age and sex effect in kw, CBF and ATT.** Brain regions with significant age×sex effects in kw, CBF and ATT with corresponding locations (AAL template), cluster sizes, T values and P values.

**Supplementary file 1f**

| **Brain regions** | **CBF**  **Estimate** [95% CI];  **P value** | | **ATT**  **Estimate** [95% CI];  **P value** | |
| --- | --- | --- | --- | --- |
|  | **8 - 61 years** | **62 - 92 years** | **8 - 61 years** | **62 - 92 years** |
| **Gray matter** | **0.03** [-0.30, 0.35]; **0.88** | **0.43** [-0.15, 1.01]; **0.14** | **-0.03** [-0.05 -0.01]; **0.01** | **-0.07** [-0.11, -0.03]; **<0.001** |
| **White matter** | **-0.01** [-0.39, 0.37]; **0.97** | **0.31** [-0.32, 0.93]; **0.33** | **-0.02** [-0.05, 0.00]; **0.08** | **-0.07** [-0.11, -0.03]; **<0.001** |
| **Frontal lobe** | **-0.03** [-0.35, 0.30]; **0.87** | **0.18** [-0.42, 0.77]; **0.56** | **-0.02** [-0.05, 0.00]; **0.08** | **-0.07** [-0.11, -0.03]; **<0.001** |
| **Temporal lobe** | **0.09** [-0.23, 0.40]; **0.58** | **0.51** [-0.03, 1.04]; **0.06** | **-0.03** [-0.05, -0.01]; **0.01** | **-0.05** [-0.08, -0.01]; **<0.01** |
| **Parietal lobe** | **0.15** [-0.23, 0.53]; **0.44** | **0.40** [-0.23, 1.03]; **0.21** | **-0.03** [-0.05, 0.00]; **0.053** | **-0.03** [-0.08, 0.02]; **0.20** |
| **ACC** | **-0.20** [-0.56, 0.15]; **0.26** | **0.21** [-0.44, 0.85]; **0.52** | **-0.01** [-0.04, 0.01]; **0.33** | **-0.05** [-0.09, -0.01]; **0.007** |
| **PCC** | **0.17** [-0.19, 0.54]; **0.35** | **0.46** [-0.07, 0.98]; **0.09** | **-0.03** [-0.06, 0.00]; **0.10** | **-0.03** [-0.07, 0.01]; **0.09** |
| **Precuneus** | **0.03** [-0.37, 0.42]; **0.90** | **0.32** [-0.29, 0.92]; **0.30** | **-0.03** [-0.06, -0.00]; **0.03** | **-0.03** [-0.07, 0.01]; **0.16** |
| **Caudate** | **-0.45** [-0.93, 0.03]; **0.06** | **0.45** [-0.40, 1.30]; **0.29** | **-0.02** [-0.04, 0.01]; **0.31** | **-0.05** [-0.09, -0.01]; **0.007** |
| **Putamen** | **-0.54** [-1.01, -0.08]; **0.02** | **0.17** [-0.49, 0.82]; **0.61** | **0.00** [-0.03, 0.02]; **0.86** | **-0.05** [-0.08, -0.01]; **0.006** |
| **Amygdala** | **-0.57** [-0.98, -0.17]; **0.006** | **0.06** [-0.60, 0.71]; **0.87** | **0.01** [-0.02, 0.03]; **0.64** | **-0.06** [-0.09, -0.02]; **0.002** |
| **Hippocampus** | **-0.59** [-0.97, -0.22]; **0.002** | **0.15** [-0.55, 0.85]; **0.67** | **0.00** [-0.03, 0.02]; **0.80** | **-0.07** [-0.10, -0.03]; **<0.001** |
| **PHG** | **-0.50** [-0.85, -0.16]; **0.004** | **-0.03** [-0.66, 0.61]; **0.94** | **-0.01** [-0.03, 0.02]; **0.50** | **-0.07** [-0.11, -0.03]; **<0.001** |
| **MTL** | **-0.54** [-0.90, -0.18]; **0.003** | **0.09** [-0.59 0.77]; **0.79** | **-0.00** [-0.03, 0.02]; **0.65** | **-0.07** [-0.11, -0.04]; **<0.001** |

**Supplementary file 1f: Association between kw and CBF or ATT in 14 brain regions for participants aged between 8 - 61 years and 62 - 92 years.** Linear regressions incorporating sex as a covariate were conducted, and the estimated coefficients are presented with 95% confidence intervals (CI) and P values.
